# Using Different Ions to Tune Graphene Stack Structures from Sheet- to Onion-Like During Plasma Exfoliation, with Supercapacitor Applications

**DOI:** 10.1186/s11671-019-2963-5

**Published:** 2019-04-23

**Authors:** Po-Jen Yen, Sumanta Kumar Sahoo, Ya-Chi Chiang, Shih-Yu Huang, Chia-Wei Wu, Yung-Chi Hsu, Kung-Hwa Wei

**Affiliations:** Department of Materials Science and Engineering, National Chiao Tung University, Hsinchu, 30010 Taiwan

**Keywords:** Graphene sheet and onion structures, Plasma, Cathodic exfoliation, Supercapacitor

## Abstract

In this article, we report a facile and simple approach for tuning graphene nanosheet structures (GNS) with different ions in the electrolytes through cathodic plasma exfoliation process in electrochemical reactions. We obtained sheet- and onion-like GNS when aqueous electrolyte NaOH and H_2_SO_4_, respectively, were present during plasma exfoliation in the electrochemical reactions, as evidenced from scanning electron microscopy and transmission electron microscopy images. Moreover, the onion-like GNS exhibited a specific surface area of 464 m^2^ g^−1^ and a supercapacitive performance of 67.1 F g^−1^, measured at a scan rate of 5 mV s^−1^ in 1 M NaCl; these values were much higher than those (72 m^2^ g^−1^ and 21.6 F g^−1^, respectively) of the sheet-like GNS. This new approach for efficiently generating tunable stacked graphene structures with different ions, in the cathodic plasma exfoliation process, has promising potentials for use in energy storage devices.

**Electronic supplementary material:**

The online version of this article (10.1186/s11671-019-2963-5) contains supplementary material, which is available to authorized users.

## Introduction

With the rapid depletion of fossil fuels and the rising controversy over their use, the development of energy storage and conversion devices—including batteries [1, 2] and supercapacitors [3, 4]—is becoming increasingly necessary for the global community. Supercapacitors have been studied extensively because of the excellent power densities and cycling lifes. They can be divided into two types—pseudocapacitors and electrical double-layer capacitors (EDLCs)—based on their charge storage mechanisms. In pseudocapacitors, the charge storage depends on reversible Faraday reactions, for which the electrodes are often made of transition metal oxides and conducting polymers. For EDLCs, the electrical energy is stored through ion adsorption and desorption processes, using typical electrode materials (e.g., porous carbon materials). In recent years, two-dimensional (2D) materials, including graphene and transition metal dichalcogenides (TMDCs), have attracted considerable attention for their excellent physical and chemical properties. TMDCs of the type MX_2_, where *M* is a transition metal atom (e.g., Mo, W) and *X* is a chalcogen atom (S, Se, or Te), possess a sandwich-like structure and have adequate theoretical capacitance and catalytic activity [5–7]. Graphene, a 2D single-layered carbon material having honeycomb-like lattices, has also drawn much attention for its extraordinary electrical [8, 9] and thermal conductivities [10], elasticity [11, 12], transparency [13–15], and specific surface area [16].

Two forms of graphene are common: thin films and powders. Mechanical exfoliation [17] and epitaxial [18, 19] and chemical vapor deposition [20–23] can be used to produce high-quality graphene thin films—displaying flexibility and high transparency—that can be applied in electrical devices, but high costs and careful control are required, due to the necessary production equipment and precursors. Powdery graphene is more widely used because of its greater mass-production, superior stability, large specific surface area, and high applicability to charge storage applications (e.g., capacitors [24], lithium-ion batteries [25, 26]). Many methods—including ball-milling, arc-discharging, and solution plasma and electrochemical exfoliation—have been reported for the production of powdery graphene materials. The ball-milling and arc-discharging methods involve physical exfoliation and can be used for industrial-scale production [27, 28]; nevertheless, expensive equipment and vacuum conditions are essential, resulting in higher costs. The solution plasma and electrochemical exfoliation methods can both generate graphene sheets from graphite electrodes using simple setups and readily accessible electrolytes [29, 30]. Although solution plasma exfoliation requires high voltages (> 1000 V) between very close anodes and cathodes and, therefore, extra care, electrochemical exfoliation can be performed at relatively lower applied voltages (< 20 V), but higher oxidation levels are introduced into the products, due to anodic exfoliation during electrolysis. Furthermore, the ready re-aggregation of layer-like graphene, through strong π-stacking interactions, can weaken its performance.

Several methods have been developed to improve the performance of active materials, including composite methods for energy storage devices [31–33], single-atom-confined 2D materials and surface regulation for catalysis [34, 35], heterostructures in photovoltaics [36], and structural engineering for further technological applications [37]. These methods all result in synergistic effects of the heterogeneous materials, with more accessible active sites provided by heterojunctions and the larger surface areas of the modified materials. For graphene materials, sacrificial-template methods and aerogel fabrication are the most common processes used to overcome the issues of layered re-aggregation. The sacrificial-template methods normally employ aqueous solutions of graphene oxide and polymer microspheres that are subjected to high-temperature reduction, often yielding graphene materials of high specific surface areas [38, 39]; nevertheless, the blending uniformity must be controlled well in terms of the nature of the surface charge, and such methods are time-consuming. Graphene aerogels can be synthesized at low temperatures [40–42] or under ambient conditions [43] to give outstanding specific surface areas; freeze gelation and solvent sublimation are, however, time-consuming and require toxic solvents to preserve the porous architectures of the aerogels. Hence, the opportunity remains to develop an easy-to-setup, inexpensive, one-step, and highly efficient method for the production of powdery graphene of high specific surface area under ambient conditions without using hazard chemicals.

Here, we report a facile method for regulating the morphologies of as-produced graphene nanosheet structures (GNS) through the use of various electrolytes during electrochemical cathodic plasma exfoliation under ambient conditions. Within a short time, we could produce GNS having high specific surface areas. We propose a mechanism for the cathodic plasma exfoliation and discuss the morphologies, structures, and supercapacitive performances of the GNS when used as electrode materials.

## Methods

### Materials

Sodium hydroxide and sulfuric acid were purchased from Sigma–Aldrich. Graphite rods (*φ* 7 mm; length 70 mm) and graphite (99.5%) were obtained from Toyo Tanso (Taiwan). Platinum plates (length 200 mm; width 30 mm; thickness 0.7 mm) were purchased from Guang Yi Eleciron Chemical Equipment (Taiwan). The GNS samples were prepared by the following procedures: a graphite rod and a platinum plate were used as the cathode and anode, respectively, during the exfoliation process; they were immersed at depths of 10–20 and 60–120 mm, respectively, under the electrolyte. Aqueous 2 M H_2_SO_4_ and 4 M NaOH were used as electrolytes. Once a potential of 60 V was applied by the power supply (LinVac Tech, Taiwan), the graphite electrode was covered by the cathodic plasma and GNS were immediately exfoliated into the electrolyte. After vacuum filtration using a nylon filter (Millipore; pore size 0.2 μm), the as-produced samples were washed and dried at 70 °C for 3 h. After dispersing the GNS in ethanol at a concentration of 0.3 mg mL^−1^, the dispersion was subjected to centrifugation (2000 rpm, 30 min) to remove any un-exfoliated graphite flakes. The supernatant was subjected to further characterizations. The GNS obtained using H_2_SO_4_ and NaOH are denoted herein as GNS_H^+^ and GNS_Na^+^, respectively.

### Characterization

The microstructures of the GNS samples were investigated using cold field emission scanning electron microscopy (SU-8010, Hitachi). A spherical aberration-corrected scanning transmission electron microscope (ARM200F, JEOL) was used to obtain transmission electron microscopy (TEM), high-resolution TEM (HRTEM), and fast Fourier transform (FFT) images. HRTEM was further used to determine the thickness distribution of the GNS samples. Raman spectra for determining the defects in the GNS samples were obtained using a Raman spectrometer (HORIBA, LabRAM HR) with a He/Ne laser source (laser excitation wavelength 632.8 nm); N_2_ gas adsorption/desorption isotherms were recorded using the Brunauer–Emmett–Teller (BET) method (ASAP 2020, Micromeritics). Pore size distributions were determined using the Barret–Joyer–Halenda (BJH) model and the desorption branches of the isotherms. X-ray photoelectron spectroscopy (XPS; ULVAC, PHI Quantera SXM) and an Al X-ray source (1200 eV) were used to analyze the surface compositions of the as-produced GNS. A D2 X-ray diffractometer (Bruker), equipped with a Cu Kα tube and a Ni filter (*λ* = 0.1542 nm), was used for structural analysis of graphite and the as-produced GNS.

### Electrochemical Measurements

The electrochemical performances of graphite, GNS_H^+^, and GNS_Na^+^ were determined in 1 M NaCl, using a Zahner Zennium electrochemical workstation in a three-electrode mode, including a standard calomel electrode as the reference electrode and a platinum foil as the counter electrode. The testing was conducted in the potential range of − 0.4 to + 0.6 V. Each electrode was prepared by mixing 90 wt% of the sample and 10 wt% of poly (vinylidene fluoride) with *N*-methylpyrrolidone (NMP) and then coating the slurry onto graphite papers and drying at 80 °C overnight. The specific capacitance (*C*, F g^−1^) can be calculated from cyclic voltammetry (CV) curves using the equation:

$$ C=\frac{\int \mathrm{Idv}}{2\times v\times \Delta  m\times \Delta  V\ } $$

where *v* is the scan rate (mV s^−1^), *∆m* is the mass of the active material, *∆V* represents the potential window, and Idv is the area under the CV curve (Q). Electrochemical impedance spectroscopy (EIS) was also conducted using an amplitude of the alternating voltage of 5 mV; data were collected in the frequency range from 100 mHz to 100 kHz.

## Results and Discussion

**Fig. 1:**
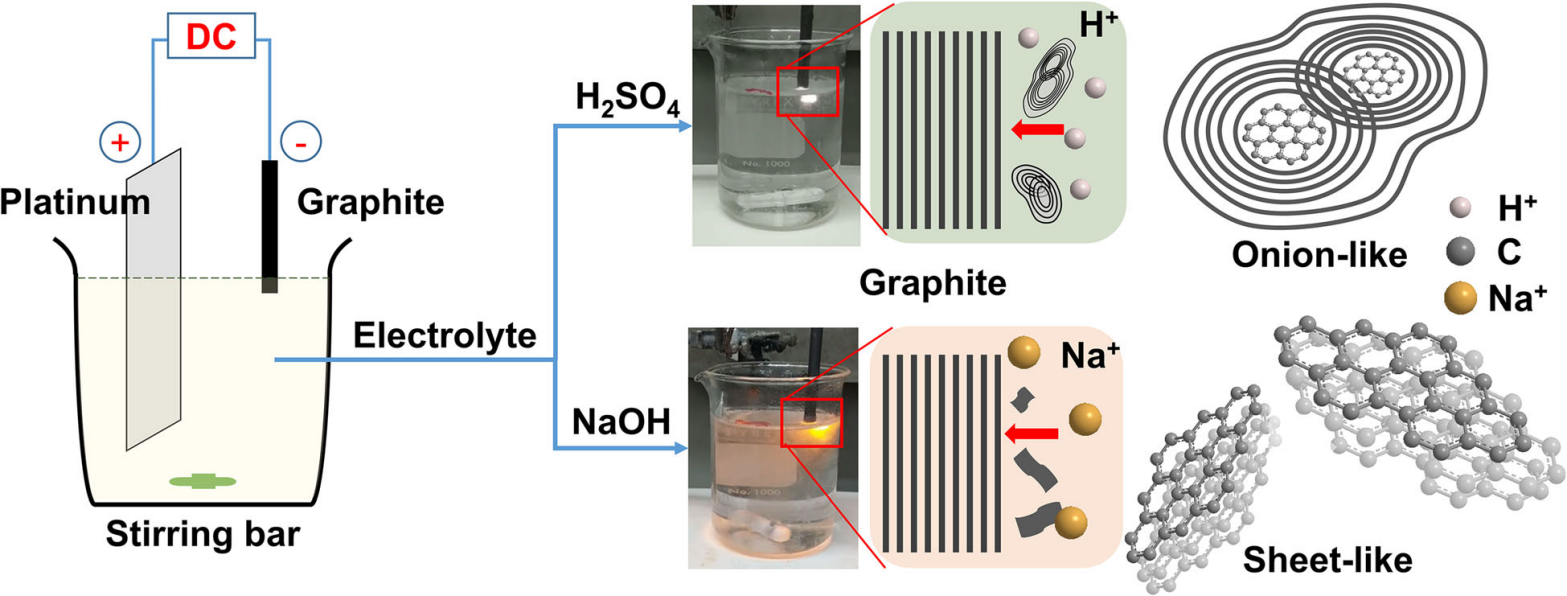
Schematic representations of the experimental setup and the cathodic plasma exfoliation processes using NaOH and H_2_SO_4_ as electrolytes, along with digital images of the experiments

Figure 1 presents schematic and digital images of the cathodic plasma exfoliation setup and schematic images of the exfoliation process and the as-produced materials obtained using aqueous NaOH and H_2_SO_4_ electrolytes. A graphite rod was used as the cathode, while a platinum plate was used as the anode. In contrast to traditional electrochemical exfoliation [44, 45], here the ratio of the surface area in contact with the electrolytes between the cathode and anode was rather small (1:10), such that a higher current density could be created at the submerged part of the cathode [46, 47]. With rapid heat accumulation arising from the higher current density, the vaporization of water at the cathode initially dominated over the H_2_ gas produced from the normal electrolysis. When the voltage difference reached 60 V, the immersed part of the graphite was promptly covered by a stable and thin gas sheath, accompanied by a plasma, resulting in immediate exfoliation of graphene sheets [47, 48]. Because of the cations and water in the electrolytes, orange and white plasmas formed when using the aqueous NaOH and H_2_SO_4_ electrolytes, respectively. In previous studies [49, 50], we found that sheet-like graphene nanosheets could be generated through cathodic plasma exfoliation when using NaOH or KOH as the electrolyte. In this present study, however, we obtained an onion-like morphology when the electrolyte was H_2_SO_4_, presumably because of a different type of cation bombardment during cathodic plasma exfoliation [51]. We discuss the mechanisms affecting the as-produced morphologies along with their materials characterization below.

**Fig. 2:**
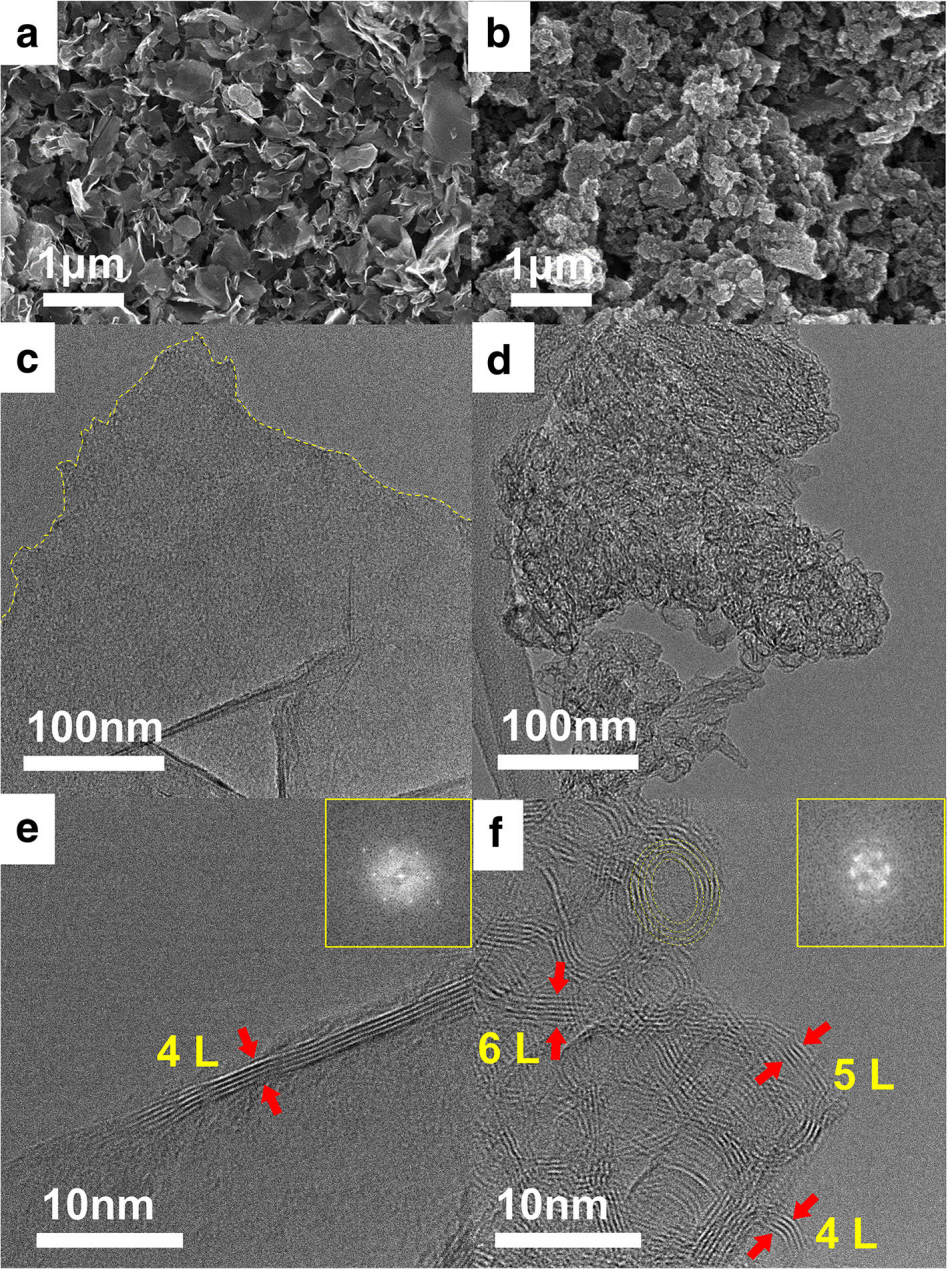
**a**, **b** FESEM images of **a** GNS_Na^+^ and **b** GNS_H^+^; **c**, **d** bright-field TEM images and **e**, **f** HRTEM images with corresponding FFT images of **c**, **e** GNS_Na^+^ and **d**, **f** GNS_H^+^

Figure 2a presents FESEM images of GNS_Na^+^ at a magnification of 20 k; the sheet-like morphology indicates the successful exfoliation of typical graphene sheets from the cathodic plasma process, due to thermal stress and potential-driven ion bombardment. Interestingly, Fig. 2b displays a broccoli-like morphology with bunches on the surfaces of GNS_H^+^ material. We suspect that this morphological difference was related to different ion bombardment effects during the cathodic plasma exfoliation: the smaller H^+^ ions not only exfoliated the graphene sheets from the graphite electrode but also decorated the surfaces to create nanostructures, while the larger Na^+^ ions had only an exfoliating effect [50, 52]. Figure 2c and d display TEM images of GNS_Na^+^ and GNS_H^+^, respectively. GNS_Na^+^ had a typical graphene morphology with a smooth layer-like surface and fractured edges; in contrast, GNS_H^+^ featured multiple stacked nanostructures. HRTEM revealed that the as-produced GNS_Na^+^ (Fig. 2e) comprised four layers of graphene sheets; an onion-like morphology existed for GNS_H^+^ (Fig. 2f), with diameters from 4 to 10 nm. Interestingly, the rings comprised four to six layers of graphene sheets. The spherical onion-like structure presumably resulted from the effect of H^+^ ions during the bombardment, consistent with the FESEM data. Compared with the larger Na^+^ ions, the smaller H^+^ ions facilitated more thorough bond-breaking and dissociation of the graphene radicals produced during plasma exfoliation [53, 54]. As soon as these radical species were sputtered outward from the plasma zone to the electrolyte, they were quenched because of the temperature gradient [55]; at the same time, due to the energy loss of the highly energized radicals, they recombined to form the spherical onion-like structure to minimize the surface energy. The insets to Fig. 2e and f present FFT images of GNS_Na^+^ and GNS_H^+^, respectively; both reveal typical hexagonal diffraction patterns, suggesting that graphene lattices remained after cathodic plasma exfoliation. Because of the many diffraction spots arising from the multiple crystal orientations of the onion-like graphene, additional ring patterns were evident in the image from GNS_H^+^. We could only roughly control the thickness of the as-produced graphene when varying the ions in the electrolytes, owing to the heterogeneous nature of ion intercalation and bombardment [50, 56, 57]. Additional file 1: Figure S1a reveals the thickness distributions of more than 30 GNS samples, as determined from their HRTEM images. Notably, GNS_H^+^ and GNS_Na^+^ samples having less than six graphene layers made up approximately 87 and 74%, respectively, of all the graphene nanosheets, while the percentage of graphene nanosheets comprising three to six graphene layers was larger for GNS_H^+^ than it was for GNS_Na^+^, suggesting that the uniformity of the as-produced GNS was associated with the type of ions: the smaller H^+^ ions could induce more bond-breaking and exfoliation than could the Na^+^ ions during the electrochemical plasma process. We performed two additional sets of experiments to examine the reproducibility of the onion-like GNS produced by H^+^ ions. Additional file 1: Figures S1b and S1c reveal the histograms of GNS_H^+^ from the second and third batches; the thickness distributions were similar to those in the first batch, with the HRTEM images of the onion-like morphologies depicted in the insets to Additional file 1: Figures S1b and S1c, suggesting the consistent reproducibility of GNS_H^+^.

**Fig. 3:**
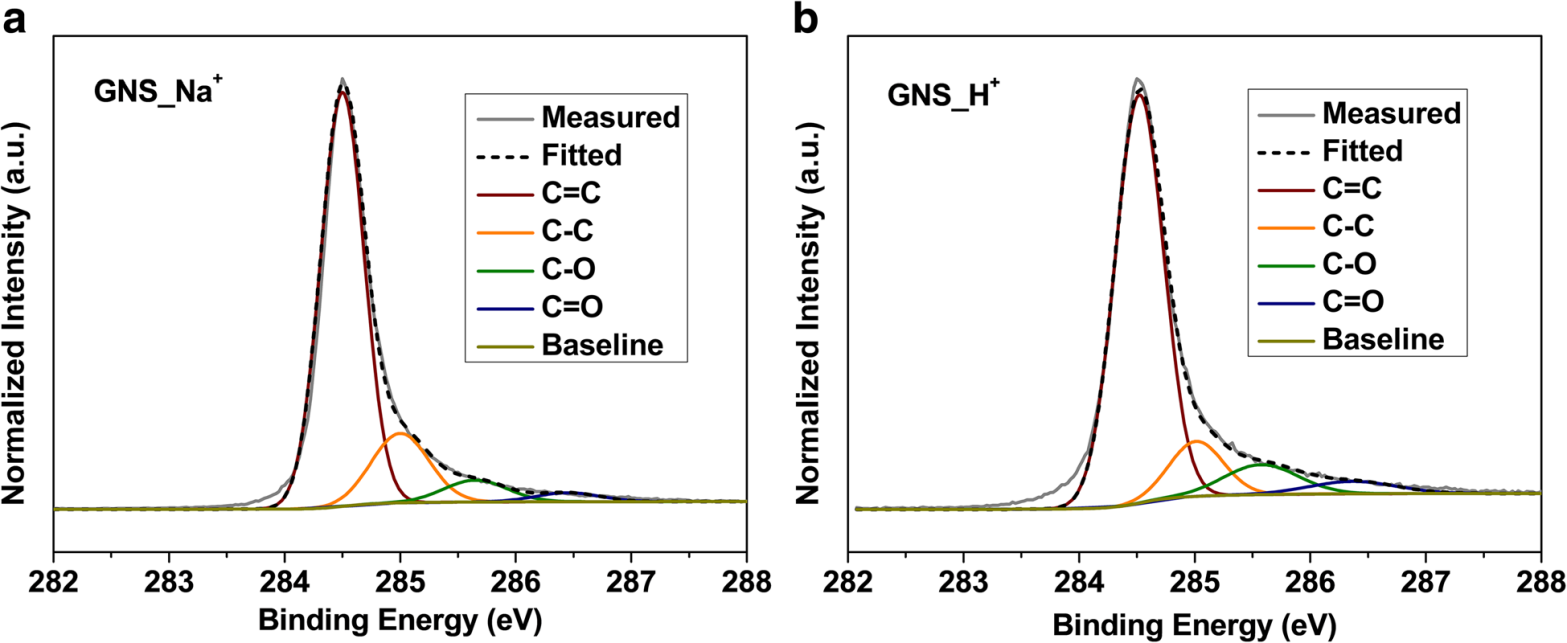
High-resolution C 1s XPS spectra of **a** GNS_Na^+^ and **b** GNS_H^+^

We used XPS to investigate the surface chemical composition of the as-produced GNS. Additional file 1: Figure S2a displays the survey spectra of both samples, revealing the presence of carbon and oxygen species. The oxidation of GNS can be ascribed to the cathodic plasma exfoliation, which radicalized the graphene and electrolytes with water molecules. After radical reactions, quenching, and atomic rearrangement, oxygen-containing functional groups were created at the surfaces of the as-produced materials. Figure 3a and b present deconvoluted C 1s spectra of GNS_Na^+^ and GNS_H^+^, respectively. In both spectra, we assign the peaks located at 284.5 and 285.1 eV to the bonding of sp^2^- and sp^3^-hybridized carbon atoms—namely C=C and C–C, respectively [58, 59]. Peaks with higher binding energies of 285.7 and 287.1 eV are referenced to C–O and C=O units. The atomic percentages of carbon and oxygen for GNS_Na^+^ were 97.9 and 2.1 at.%, respectively; GNS_H^+^ had a higher oxygen percentage (up to 6.1 at.%). The higher degree of oxidation for GNS_H^+^ presumably resulted from the extra nanostructures and defects introduced by the relatively smaller H^+^ ions—a consequence of more radicalized sites appearing on the graphene during exfoliation.

**Fig. 4:**
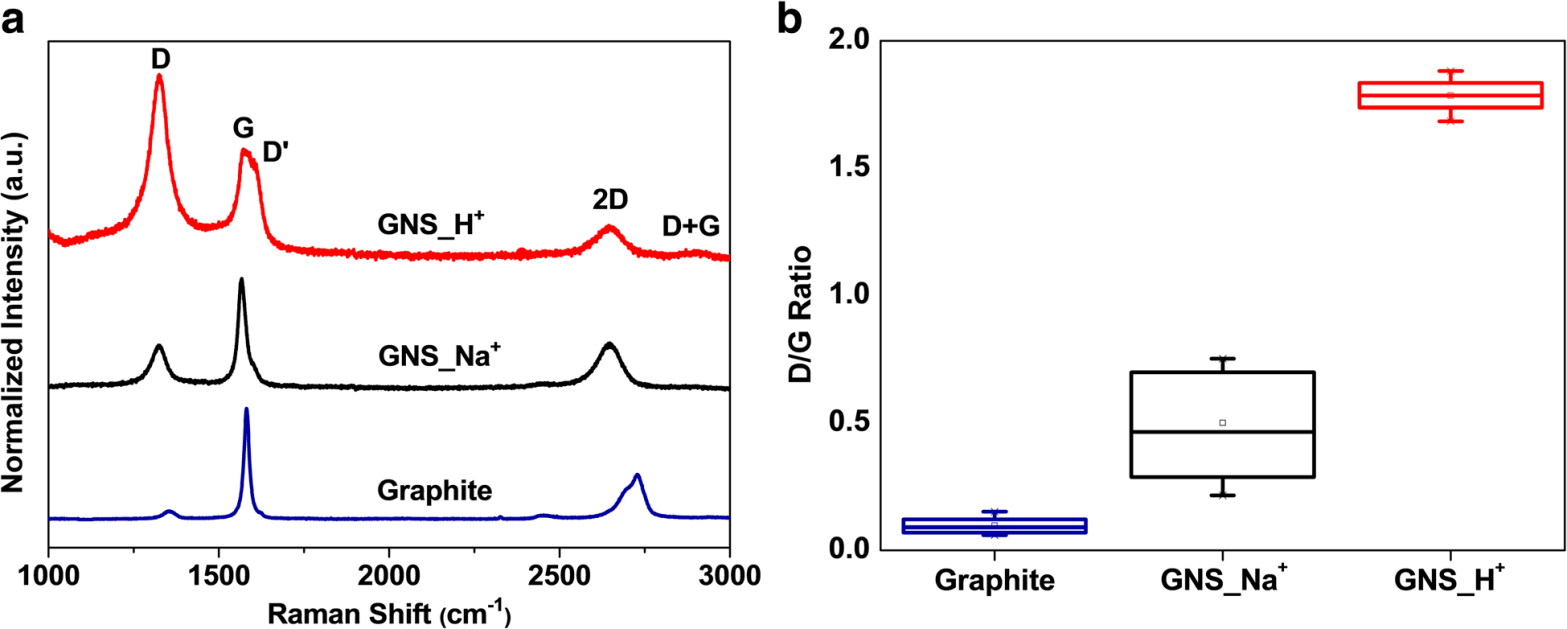
**a** Raman spectra and **b** box charts of the intensity ratios of the D- and G-bands of graphite, GNS_Na^+^, and GNS_H^+^

**Fig. 5:**
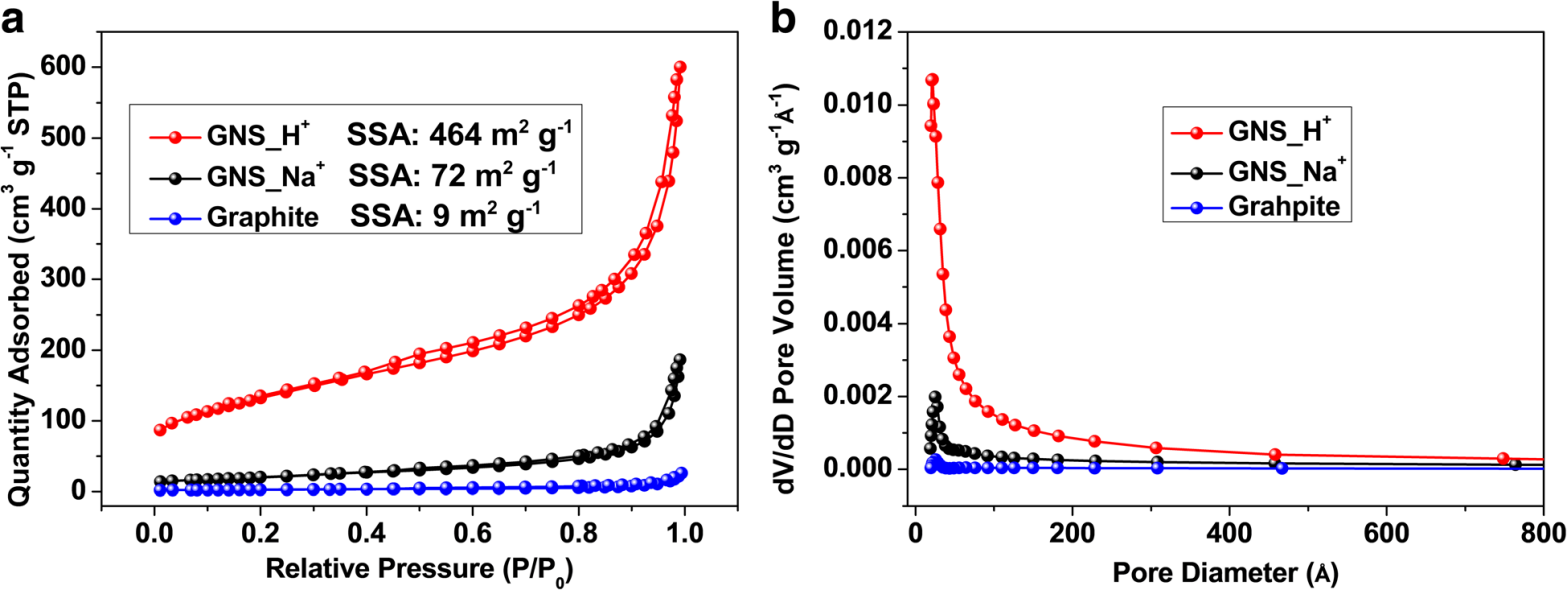
**a** Typical N_2_ adsorption/desorption isotherms and **b** BJH pore size distribution of graphite, GNS_Na^+^, and GNS_H^+^

We obtained further structural information using Raman spectroscopy. In Fig. 4a, the three distinguishing peaks at 1325, 1571, and 2648 cm^−1^ represent the D-, G-, and 2D-bands, respectively [60–63]. The D-band represents the A_1g_ mode, related to defects and structural disorder; the G-band is associated with in-plane bonding of a stretching pair formed from sp^2^-hybridized carbon atoms, as is the E_2g_ mode; related to the splitting of phonon bands or electron bands, the 2D-band is the secondary order of the D-band. The ratios of the peak intensities *I*_D_/*I*_G_ for graphite, GNS_Na^+^, and GNS_H^+^ were 0.08, 0.46, and 0.79, respectively. Because the cathodic plasma process enhanced the number of functional groups and edges and increased the structural disorder of the carbon lattice, the defect intensities of GNS_Na^+^ and GNS_H^+^ were higher than that of graphite. Furthermore, we ascribe the higher value of *I*_D_/*I*_G_ for GNS_H^+^ to the greater levels of oxidation and nanostructure formation, as evidenced using XPS, FESEM, and TEM. The relatively distinguishable D′ (1610 cm^–1^) and D + G (2909 cm^–1^) bands confirmed the greater disorder of GNS_H^+^. Because of van der Waals forces, some agglomeration occurred in the powdery GNS samples, as evidenced from the FESEM images; from our Raman spectra in Fig. 4a, the symmetrical and downshifted 2D-bands of GNS_Na^+^ and GNS_H^+^, relative to that of graphite, suggest that the few-layer graphene structures were mostly retained without large degrees of restacking to form graphite. Figure 4b also provides box charts of the intensity ratios of the D- and G-bands for GNS_Na^+^ and GNS_H^+^, each collected from 15 samples. GNS_H^+^ featured higher defect levels on average, suggesting that most of the crystallinity domains in the onion-like morphology were smaller than those of the sheet-like GNS_Na^+^. Interestingly, the statistical range of values of *I*_D_/*I*_G_ for GNS_H^+^ was narrower than that for GNS_Na^+^, indicating that GNS_H^+^ had superior uniformity, associated with the smaller H^+^ ions inducing more thorough bond-breaking and dissociation of graphene radicals. The XRD patterns of the graphite, GNS_Na^+^, and GNS_H^+^ reveal (Additional file 1: Figure S2b) additional structural information. The pattern for graphite featured a sharp diffraction peak at 26.7°, which we assigned to the (002) diffraction, indicating a high degree of graphitization and an interlayer distance 0.334 nm; this peak for both GNS was downshifted by 0.1°, consistent with an increase in their interlayer distances. The full widths at half maximum (FWHMs) of the X-ray diffraction peaks near 26.7° for graphite, GNS_Na^+^, and GNS_H^+^ were 0.11, 0.40, and 2.7°, respectively, suggesting that the crystal sizes decreased in that order and, thus, that the amorphous nature of the GNS samples increased after cathodic plasma exfoliation. We performed N_2_ adsorption experiments to determine the BET surface areas and BJH pore sizes, with distributions, of the samples. Figure 5a reveals that the N_2_ adsorption/desorption isotherms had typical H3 hysteresis loops, as classified by the International Union of Pure and Applied Chemistry (IUPAC). The hysteresis loops appeared at relatively low pressure (0.4–0.8), indicating the presence of mesopores in the as-produced materials [50]. The specific surface areas of graphite, GNS_Na^+^, and GNS_H^+^ were 9, 72, and 464 m^2^ g^−1^, respectively. The higher specific surface area of GNS_H^+^ is consistent with its onion-like nanostructures, confirming the participation of H^+^ ions during the cathodic exfoliation. Theoretically, a higher surface area for graphene as an electrode material would provide more absorption sites for ions during electrochemical measurements. The BJH pore size distributions of the samples (Fig. 5b) revealed that the dominant pore sizes in the range 2–20 nm for graphite, GNS_Na^+^, and GNS_H^+^ were 2.5, 14.9, and 9.2 nm, respectively. Thus, the dominant pore size of the as-produced GNS was smaller when fabricated using H^+^ ions. Nevertheless, the pore volume of GNS_H^+^ (0.928 cm^3^ g^−1^) was larger than those of GNS_Na^+^ (0.289 cm^3^ g^−1^) and graphite (0.058 cm^3^ g^−1^), suggesting that the porous structures between the nano-bunches of GNS_H^+^ provided extra spaces for access of the electrolyte and for ion transport during electrochemical charging and discharging.

**Fig. 6:**
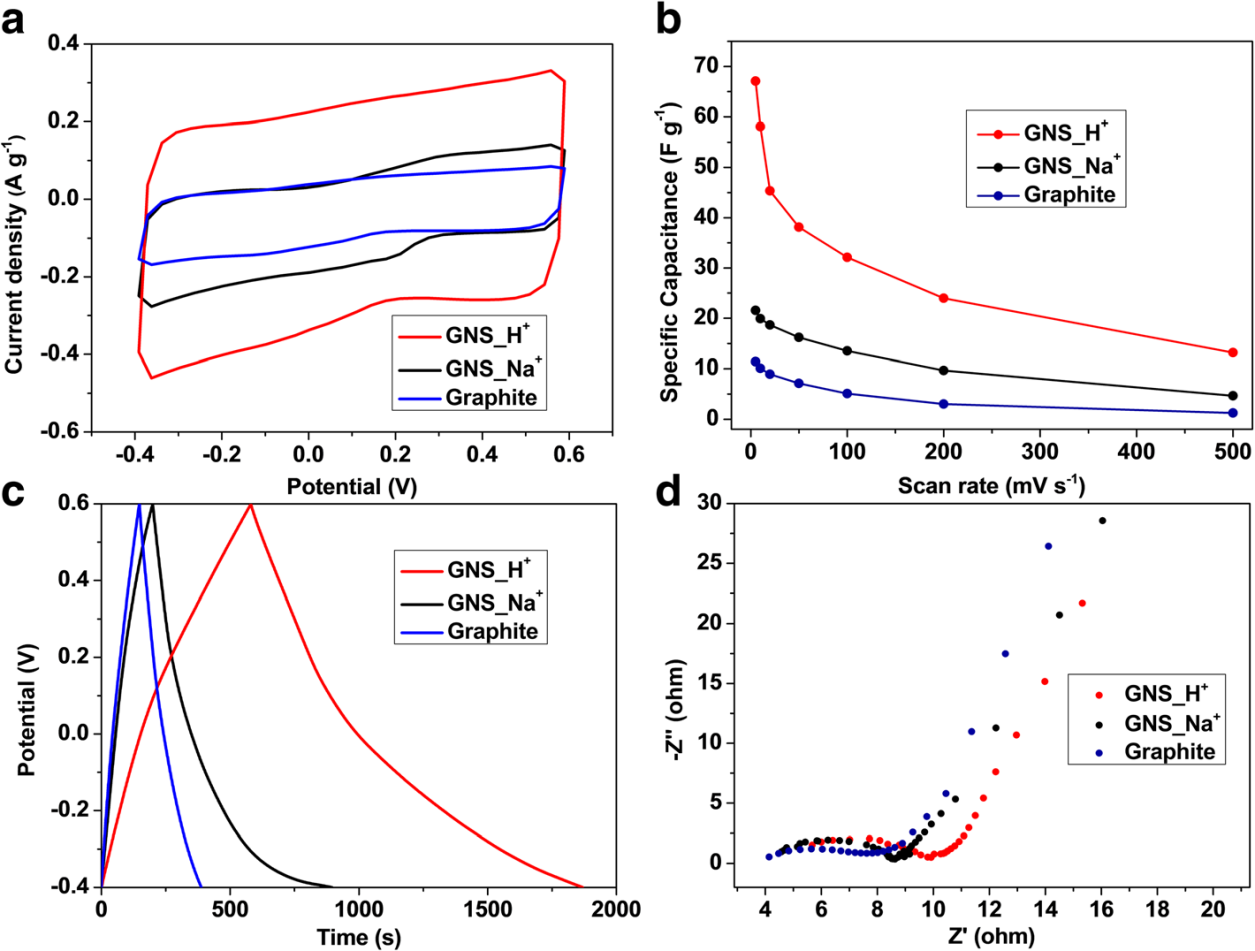
**a** Cyclic voltammograms of the various samples, recorded at 5 mV s^−1^. **b** Specific capacitances of the samples, determined at scan rates from 5 to 500 mV s^−1^. **c** Galvanostatic charge/discharge curves recorded at 0.1 A g^−1^. **d** Nyquist plots of the samples measured from 100 mHz to 100 kHz; inset: corresponding expanded high-frequency region of the plots

We used a three-electrode system to determine the electrochemical properties of graphite, GNS_Na^+^, and GNS_H^+^ in 1 M NaCl at potentials from − 0.4 to + 0.6 V. Figure 6a presents the cyclic voltammograms of graphite, GNS_Na^+^, and GNS_H^+^ recorded at a scan rate of 5 mV s^−1^. We observe quasi-rectangular shapes of the measured curves with no obvious Faradaic reaction, suggesting that the electrode materials had excellent electrical double layer capacitance as a result of highly accessible ions [64]. Table 1 compares the specific surface areas, pore volumes, specific capacitances, and charge-transfer resistances of graphite, GNS_Na^+^, and GNS_H^+^. The specific capacitances, determined at a scan rate of 5 mV s^−1^, for graphite, GNS_Na^+^, and GNS_H^+^ were 11.4, 21.6, and 67.1 F g^−1^, respectively. Because graphene structures of only a few layers were produced during the cathodic plasma exfoliation, the supercapacitive performances of both GNS were higher than that of the graphite; furthermore, the 3D-structured onion-like GNS_H^+^ had even higher specific capacitance because its larger specific surface area and pore volume could accommodate extra ions during charging and discharging. Additional file 1: Table S1 compares the synthesis methods, times, and temperatures; the specific surface areas; and the electrochemical performances (in NaCl solutions at different scan rates) of various graphene materials that have been reported in the literature. Although the specific capacitances in this present study are lower than some of those reported previously, most other synthesis methods have required longer processing times and higher temperatures to produce graphene materials with high specific surface areas. Hence, we believe that our facile, one-step, and green cathodic plasma exfoliation process is very competitive at producing good-quality GNS. Additional file 1: Figures S3a–c present the cyclic voltammograms of graphite, GNS_Na^+^, and GNS_H^+^ recorded at various scan rates. Upon increasing the scan rate, the measured curves of each sample retained their quasi-rectangular shapes, suggesting that the electrode materials had excellent electrical double-layer capacitance without obvious Faradaic reaction. Notably, both GNS exhibited excellent electrochemical stability over a wide range of scan rates; the distinct enhancements in current density upon increasing the scan rate suggest superior rate abilities for both of these electrode materials. Figure 6b reveals that the specific capacitances of GNS_H^+^ were relatively higher than those of GNS_Na^+^ at all scan rates from 5 to 500 mV s^−1^, presumably because of the higher specific surface area arising from the onion-like nanostructures of GNS_H^+^. Figure 6c displays the typical galvanostatic charge/discharge curves of the graphene materials GNS_Na^+^ and GNS_H^+^ at a current density of 0.1 A g^−1^. Their highly symmetric triangular curves imply that both samples underwent no obvious potential (iR) drops, indicating low internal resistances within these electrode materials. Additional file 1: Figures S3d–f presents the galvanostatic charge/discharge curves of the samples at various current densities. The measured curves retain the notable triangular shapes in their symmetry upon increasing the current density, indicating the outstanding rate abilities for these as-produced GNS with different morphologies. We measured the cycling abilities of GNS_Na^+^ and GNS_H^+^ over 1000 cycles at a scan rate of 100 mV s^−1^ (Additional file 1: Figure S4). Every material displayed outstanding capacitance retention: 93, 91, and 88% for graphite, GNS_Na^+^, and GNS_H^+^, respectively. The slightly lower capacitance retention percentage of GNS_H^+^ presumably arose from non-reversible ion trapping in the smaller pores of its nanostructures during the charging and discharging processes. EIS is a powerful method for measuring the electrical conductivities of carbon electrodes. Figure 6d displays the Nyquist plots of graphite, GNS_Na^+^, and GNS_H^+^ measured in the frequency range from 100 mHz to 100 kHz. In general, the first intercept to the real axis of the measured curves in the high-frequency region, known as the electrochemical series resistance (*R*_s_), can be related to the ionic conductivity of the electrolyte in the electrode materials, the intrinsic resistance of the electrode materials, and the contact resistance of the materials to the current collector. Small values of *R*_s_ were evident for both of our samples in their measured curves, indicating the superior conductivity of these as-produced graphene materials. The impedance plot also featured semicircles in the high- and medium-frequency regions, related to charge-transfer processes at the interfaces between the active materials on the electrode and the electrolyte; a 45° inclined line in the low-frequency region suggested Warburg impedance correlated to mass transport [65, 66]. The charge-transfer resistances (*R*_ct_) of graphite, GNS_Na^+^, and GNS_H^+^ were 3.5, 3.9, and 4.6 Ω, respectively, revealing highly conductive graphite and graphene materials that facilitated ionic diffusion during charging and discharging. The slightly greater value of *R*_ct_ for GNS_H^+^ can be correlated to its smaller crystallinity area and greater level of oxidation, compared with those of graphite and GNS_Na^+^. Hence, although different morphologies formed in the presence of Na^+^ and H^+^ ions, the conductivities of the GNS produced through cathodic plasma exfoliation were not compromised, due to the preservation of the crystallinity of the graphene lattice. Because of the curvature of the onion-like structures, GNS_H^+^ might have featured more structural dislocations and, thus, more active sites in the graphene hexagonal lattices in the curved basal plane [67–69]. Therefore, the ion absorption for the onion-like GNS_H^+^ could have occurred at more edges and basal plane, rather than only at the basal plane as in the case for the sheet-like GNS_Na^+^. Moreover, the higher oxidative state of GNS_H^+^ could provide a more hydrophilic surface for interaction with the aqueous electrolyte and, thus, allow more efficient transport and accessible micro-tunnels for ions during charging and discharging, resulting in higher supercapacitive performances.

**Table 1:** Specific surface areas, pore volumes, specific capacitances, and charge transfer resistances of graphite, GNS_Na^+^, and GNS_H^+^

	Specific surface area (m^2^ g^−1^)	Pore volume (cm^3^ g^−1^)	Specific capacitance (F g^−1^) at 5 mV s^−1^	Charge-transfer resistance (*R*_ct_)
Graphite	9	0.058	11.4	3.5
GNS_Na^+^	72	0.289	21.6	3.9
GNS_H^+^	464	0.928	67.1	4.6

## Conclusion

We have produced typical sheet-like GNS when using aqueous NaOH as the electrolyte in a cathodic plasma exfoliation process performed within a short period of time under ambient conditions. When the electrolyte was replaced by aqueous H_2_SO_4_, an onion-like morphology was introduced to the as-produced GNS, which featured a superior specific surface area (464 m^2^ g^−1^) and pore volume (0.928 cm^3^ g^−1^). We suspect that the involvement of H^+^ ions facilitated more thorough bond breaking and dissociation of radical species than did the Na^+^ ions during the cathodic plasma exfoliation. Measurements of supercapacitive performance at a scan rate of 5 mV s^−1^ in 1 M NaCl indicated that the GNS featuring the onion-like nanostructures had a specific capacitance (67.1 F g^−1^) higher than that of the GNS having the sheet-like morphology (21.6 F g^−1^). Thus, it is possible to produce GNS with different morphologies and supercapacitive performances when using readily accessible electrolytes in a facile cathodic plasma exfoliation process; furthermore, the unique onion-like GNS structure, with some retained crystallinity and curvature, created by the H^+^ ions, exhibited extraordinary conductivity and a high specific surface area, suggesting greater potential (relative to that of the corresponding sheet-like GNS) for use in energy storage devices.

## Additional file

Additional file 1: **Figure S1.** (a) The thickness distribution of more than 30 GNS determined from HRTEM of GNS_Na^+^ and GNS_H^+^(the first batch); (b) and (c) are GNS_H^+^ produced from the second and the third batch of the cathodic plasma exfoliation, respectively (scale bar 4 nm). **Figure S2.** (a) XPS survey spectra of GNS_Na^+^ and GNS_H^+^; (b) XRD diffraction patterns of graphite, GNS_Na^+^, and GNS_H^+^; inset: corresponding expanded region of the plots. **Table S1.** Synthesis methods, times, and temperatures; specific surface areas; and electrochemical performances, in NaCl solution at various scan rates, of graphene materials reported in the literature. *rt room temperature. **Figure S3.** (a–c) Cyclic voltammograms and (d–f) galvanostatic charge/discharge curves of (a, d) graphite, (b, e) GNS_Na^+^, and (c, f) GNS_H^+^, recorded at various scan rates. **Figure S4.** Cycling tests of graphite, GNS_Na^+^, and GNS_H^+^ over 1000 cycles at a scan rate of 100 mV s^−1^. (DOCX 2844 kb)

## Abbreviations

BET: Brunauer–Emmett–Teller
CV: Cyclic voltammogram
EDLC: Electrical double-layer capacitors
FESEM: Field-emission scanning electron microscopy
FFT: Fast Fourier transform
FWHM: Full width at half maximum
GNS: Graphene nanosheets
GNS_H^+^: Graphene nanosheets produced using H_2_SO_4_
GNS_Na^+^: Graphene nanosheets produced using NaOH
H_2_SO_4_: Sulfuric acid
KOH: Potassium hydroxide
NaCl: Sodium chloride
NaOH: Sodium hydroxide
NMP: N-Methyl-2-pyrrolidone
TEM: Transmission electron microscopy
XPS: X-ray photoelectron spectroscopy
XRD: X-ray diffraction

## References

[1] Yen P-J, Ilango P-R, Chiang Y-C et al (2019) Tunable nitrogen-doped graphene sheets produced with in situ electrochemical cathodic plasma at room temperature for lithium-ion batteries. Mater Today Energy. 10.1016/j.mtener.2019.01.003

[2] Li M, Lu J, Chen Z, Amine K (2018). 30 years of lithium-ion batteries. Adv Mater.

[3] Wu T-H, Chang C-T, Wang C-C (2018). Few-layer graphene sheet-passivated porous silicon toward excellent electrochemical double-layer supercapacitor electrode. Nanoscale Res Lett.

[4] Zhang L, Hu X, Wang Z (2018). A review of supercapacitor modeling, estimation, and applications: a control/management perspective. Renew Sustain Energy Rev.

[5] Lu A-Y, Zhu H, Xiao J (2017). Janus monolayers of transition metal dichalcogenides. Nat Nanotechnol.

[6] Tang S-Y, Medina H, Yen Y-T (2019). Enhanced photocarrier generation with selectable wavelengths by M-decorated-CuInS_2_ nanocrystals (M = Au and Pt) synthesized in a single surfactant process on MoS_2_ bilayers. Small.

[7] Nguyen V-T, Yang T-Y, Le P-A, et al (2019) A new simultaneous exfoliation and doping process for generating MX_2_ nanosheets for electrocatalytic hydrogen evolution reaction. ACS Appl Mater Interfaces acsami. 9b01374. 10.1021/acsami.9b01374 10.1021/acsami.9b01374 30900877

[8] Buron JD, Pizzocchero F, Jepsen PU (2015). Graphene mobility mapping. Sci Rep.

[9] Pu Y-C, Chou H-Y, Kuo W-S (2017). Interfacial charge carrier dynamics of cuprous oxide-reduced graphene oxide (Cu_2_O-rGO) nanoheterostructures and their related visible-light-driven photocatalysis. Appl Catal B Environ.

[10] Fugallo G, Cepellotti A, Paulatto L (2014). Thermal conductivity of graphene and graphite: collective excitations and mean free paths. Nano Lett.

[11] Akinwande D, Brennan CJ, Bunch JS (2017). A review on mechanics and mechanical properties of 2D materials: graphene and beyond. Extrem Mech Lett.

[12] Papageorgiou DG, Kinloch IA, Young RJ (2017). Mechanical properties of graphene and graphene-based nanocomposites. Prog Mater Sci.

[13] Rafiee J, Mi X, Gullapalli H (2012). Wetting transparency of graphene. Nat Mater.

[14] Hsu C-L, Lin C-T, Huang J-H (2012). Layer-by-layer graphene/TCNQ stacked films as conducting anodes for organic solar cells. ACS Nano.

[15] Manikandan A, Lee L, Wang Y-C (2017). Graphene-coated copper nanowire networks as a highly stable transparent electrode in harsh environments toward efficient electrocatalytic hydrogen evolution reactions. J Mater Chem A.

[16] Li Z, Song B, Wu Z (2015). 3D porous graphene with ultrahigh surface area for microscale capacitive deionization. Nano Energy.

[17] Novoselov KS, Geim AK, Morozov SV (2004). Electric field effect in atomically thin carbon films. Science.

[18] deHeer WA, Berger C, Wu X (2007). Epitaxial graphene. Solid State Commun.

[19] Medina H, Huang C-C, Lin H-C (2015). Ultrafast graphene growth on insulators via metal-catalyzed crystallization by a laser irradiation process: from laser selection, thickness control to direct patterned graphene utilizing controlled layer segregation process. Small.

[20] Chang Y-H, Lin C-T, Chen T-Y (2013). Highly efficient electrocatalytic hydrogen production by MoS_*x*_ grown on graphene-protected 3D Ni foams. Adv Mater.

[21] Chen T-Y, Loan PTK, Hsu C-L (2013). Label-free detection of DNA hybridization using transistors based on CVD grown graphene. Biosens Bioelectron.

[22] Chen X, Zhang L, Chen S (2015). Large area CVD growth of graphene. Synth Met.

[23] Chen Y-Z, Medina H, Tsai H-W (2015). Low temperature growth of graphene on glass by carbon-enclosed chemical vapor deposition process and its application as transparent electrode. Chem Mater.

[24] Yang C-C, Tsai M-H, Huang C-W (2017). Carbon nanotube/nitrogen-doped reduced graphene oxide nanocomposites and their application in supercapacitors. J Nanosci Nanotechnol.

[25] Lian P, Zhu X, Liang S (2010). Large reversible capacity of high quality graphene sheets as an anode material for lithium-ion batteries. Electrochim Acta.

[26] Wang G, Shen X, Yao J, Park J (2009). Graphene nanosheets for enhanced lithium storage in lithium ion batteries. Carbon NY.

[27] Zhao W, Fang M, Wu F (2010). Preparation of graphene by exfoliation of graphite using wet ball milling. J Mater Chem.

[28] Subrahmanyam KS, Panchakarla LS, Govindaraj A, Rao CNR (2009). Simple method of preparing graphene flakes by an arc-discharge method. J Phys Chem C.

[29] Senthilnathan J, Liu Y-F, Rao KS, Yoshimura M (2015) Submerged liquid plasma for the synchronized reduction and functionalization of graphene oxide. Sci Rep 4(4395). 10.1038/srep04395 10.1038/srep04395 PMC3957132 24637779

[30] Su C-Y, Lu A-Y, Xu Y (2011). High-quality thin graphene films from fast electrochemical exfoliation. ACS Nano.

[31] Liu M-C, Xu Y, Hu Y-X (2018). Electrostatically charged MoS_2_/graphene oxide hybrid composites for excellent electrochemical energy storage devices. ACS Appl Mater Interfaces.

[32] Wang Y, Chen Z, Lei T (2018). Hollow NiCo_2_S_4_ nanospheres hybridized with 3D hierarchical porous rGO/Fe_2_O_3_ composites toward high-performance energy storage device. Adv Energy Mater.

[33] Mohan VB, Lau K, Hui D, Bhattacharyya D (2018). Graphene-based materials and their composites: a review on production, applications and product limitations. Compos Part B Eng.

[34] Alarawi A, Ramalingam V, He J-H (2019). Recent advances in emerging single atom confined two-dimensional materials for water splitting applications. Mater Today Energy.

[35] Ma Y, Gao W, Zhang Z (2018). Regulating the surface of nanoceria and its applications in heterogeneous catalysis. Surf Sci Rep.

[36] Al-Amri AM, Cheng B, He J-H (2019). Perovskite methylammonium lead trihalide heterostructures: progress and challenges. IEEE Trans Nanotechnol.

[37] Liang X, Dong R, Ho JC (2019) Self-assembly of colloidal spheres toward fabrication of hierarchical and periodic nanostructures for technological applications. Adv Mater Technol 1800541. 10.1002/admt.201800541

[38] Wang H, Zhang D, Yan T (2013). Three-dimensional macroporous graphene architectures as high performance electrodes for capacitive deionization. J Mater Chem A.

[39] Yang Z, Chabi S, Xia Y, Zhu Y (2015). Preparation of 3D graphene-based architectures and their applications in supercapacitors. Prog Nat Sci Mater Int.

[40] Zhang X, Sui Z, Xu B (2011). Mechanically strong and highly conductive graphene aerogel and its use as electrodes for electrochemical power sources. J Mater Chem.

[41] Chen W, Li S, Chen C, Yan L (2011). Self-assembly and embedding of nanoparticles by in situ reduced graphene for preparation of a 3D graphene/nanoparticle aerogel. Adv Mater.

[42] Hu H, Zhao Z, Wan W (2013). Ultralight and highly compressible graphene aerogels. Adv Mater.

[43] Lin Y, Liu F, Casano G (2016). Pristine graphene aerogels by room-temperature freeze gelation. Adv Mater.

[44] Parvez K, Wu Z-S, Li R (2014). Exfoliation of graphite into graphene in aqueous solutions of inorganic salts. J Am Chem Soc.

[45] Yu P, Lowe SE, Simon GP, Zhong YL (2015). Electrochemical exfoliation of graphite and production of functional graphene. Curr Opin Colloid Interface Sci.

[46] Sengupta SK, Singh OP (1994). Contact glow discharge electrolysis: a study of its chemical yields in aqueous inert-type electrolytes. J Electroanal Chem.

[47] VanThanh D, Chen H-C, Li L-J (2013). Plasma electrolysis allows the facile and efficient production of graphite oxide from recycled graphite. RSC Adv.

[48] Allagui A, Wüthrich R (2009). Gas film formation time and gas film life time during electrochemical discharge phenomenon. Electrochim Acta.

[49] VanThanh D, Li L-J, Chu C-W (2014). Plasma-assisted electrochemical exfoliation of graphite for rapid production of graphene sheets. RSC Adv.

[50] Yen P-J, Ting C-C, Chiu Y-C (2017). Facile production of graphene nanosheets comprising nitrogen-doping through in situ cathodic plasma formation during electrochemical exfoliation. J Mater Chem C.

[51] Allagui A, Baranova EA, Wüthrich R (2013). Synthesis of Ni and Pt nanomaterials by cathodic contact glow discharge electrolysis in acidic and alkaline media. Electrochim Acta.

[52] Wu Z-S, Ren W, Gao L (2009). Synthesis of graphene sheets with high electrical conductivity and good thermal stability by hydrogen arc discharge exfoliation. ACS Nano.

[53] Diankov G, Neumann M, Goldhaber-Gordon D (2013). Extreme monolayer-selectivity of hydrogen-plasma reactions with graphene. ACS Nano.

[54] Felten A, McManus D, Rice C (2014). Insight into hydrogenation of graphene: effect of hydrogen plasma chemistry. Appl Phys Lett.

[55] Lee H, Bratescu MA, Ueno T, Saito N (2014). Solution plasma exfoliation of graphene flakes from graphite electrodes. RSC Adv.

[56] Parvez K, Li R, Puniredd SR (2013). Electrochemically exfoliated graphene as solution-processable, highly conductive electrodes for organic electronics. ACS Nano.

[57] Wang H, Wei C, Zhu K (2017). Preparation of graphene sheets by electrochemical exfoliation of graphite in confined space and their application in transparent conductive films. ACS Appl Mater Interfaces.

[58] Bouleghlimat E, Davies PR, Davies RJ (2013). The effect of acid treatment on the surface chemistry and topography of graphite. Carbon N Y.

[59] Gupta B, Kumar N, Panda K (2017). Role of oxygen functional groups in reduced graphene oxide for lubrication. Sci Rep.

[60] Ferrari AC (2007). Raman spectroscopy of graphene and graphite: disorder, electron–phonon coupling, doping and nonadiabatic effects. Solid State Commun.

[61] Malard LM, Pimenta MA, Dresselhaus G, Dresselhaus MS (2009). Raman spectroscopy in graphene. Phys Rep.

[62] Zhang W, Lin C-T, Liu K-K (2011). Opening an electrical band gap of bilayer graphene with molecular doping. ACS Nano.

[63] Chen Y-Z, Medina H, Lin H-C (2015). Large-scale and patternable graphene: direct transformation of amorphous carbon film into graphene/graphite on insulators via Cu mediation engineering and its application to all-carbon based devices. Nanoscale.

[64] Kumar N, Huang C-W, Yen P-J (2016). Probing the electrochemical properties of an electrophoretically deposited Co_3_O_4_/rGO/CNTs nanocomposite for supercapacitor applications. RSC Adv.

[65] Gong Y, Li D, Fu Q, Pan C (2015). Influence of graphene microstructures on electrochemical performance for supercapacitors. Prog Nat Sci Mater Int.

[66] Casero E, Parra-Alfambra AM, Petit-Domínguez MD (2012). Differentiation between graphene oxide and reduced graphene by electrochemical impedance spectroscopy (EIS). Electrochem Commun.

[67] Yang Y, Tang D-M, Zhang C (2017). “Protrusions” or “holes” in graphene: which is the better choice for sodium ion storage?. Energy Environ Sci.

[68] Wan W, Wang H (2015). First-principles investigation of adsorption and diffusion of ions on pristine, defective and B-doped graphene. Mater (Basel, Switzerland).

[69] Cheng C-C, Lu A-Y, Tseng C-C (2016). Activating basal-plane catalytic activity of two-dimensional MoS_2_ monolayer with remote hydrogen plasma. Nano Energy.

