# Supplementary material for: Using Different Ions to Tune Graphene Stack Structures from Sheet- to Onion-Like During Plasma Exfoliation, with Supercapacitor Applications
Source: Nanoscale Res Lett. 2019 Apr 23;14:141. doi: 10.1186/s11671-019-2963-5 (PMC6478787; doi:10.1186/s11671-019-2963-5)
Supplement: Supplementary file 1 — Figure S1. (a) The thickness distribution of more than 30 GNS determined from HRTEM of GNS_Na+ and GNS_H+(the first batch); (b) and (c) are GNS_H+ produced from the second and the third batch of the cathodic plasma exfoliation, respectively (scale bar 4 nm). Figure S2. (a) XPS survey spectra of GNS_Na+ and GNS_H+; (b) XRD diffraction patterns of graphite, GNS_Na+, and GNS_H+; inset: corresponding expanded region of the plots. Table S1. Synthesis methods, times, and temperatures; specific surface areas; and electrochemical performances, in NaCl solution at various scan rates, of graphene materials reported in the literature. *rt room temperature. Figure S3. (a–c) Cyclic voltammograms and (d–f) galvanostatic charge/discharge curves of (a, d) graphite, (b, e) GNS_Na+, and (c, f) GNS_H+, recorded at various scan rates. Figure S4. Cycling tests of graphite, GNS_Na+, and GNS_H+ over 1000 cycles at a scan rate of 100 mV s−1. (DOCX 2844 kb) [file 11671_2019_2963_MOESM1_ESM.docx]

**Using different ions to tune graphene stack structures from sheet- to onion-like during plasma exfoliation, with supercapacitor applications**

Po-Jen Yen, Sumanta Kumar Sahoo, Ya-Chi Chiang, Shih-Yu Huang, Chia-Wei Wu, Yung-Chi Hsu, and Kung-Hwa Wei*

Department of Materials Science and Engineering, National Chiao Tung University, 30010 Hsinchu, Taiwan

* Corresponding author

Kung-Hwa Wei

E-mail address:
Po-Jen Yen ([pojenyen.mse02g@g2.nctu.edu.tw](mailto:pojenyen.mse02g@g2.nctu.edu.tw)), Sumanta Kumar Sahoo ([sumantakumarsahoo@live.com](mailto:sumantakumarsahoo@live.com)), Ya-Chi Chiang ([ya109chi@gmail.com](mailto:ya109chi@gmail.com)), Shih-Yu Huang ([ini516888@gmail.com](mailto:ini516888@gmail.com)), Chia-Wei Wu ([kagome741wg@gmail.com](mailto:kagome741wg@gmail.com)), Yung-Chi Hsu ([a0189024@gmail.com](mailto:a0189024@gmail.com)), and Kung-Hwa Wei ([khwei@mail.nctu.edu.tw](mailto:khwei@mail.nctu.edu.tw))

**
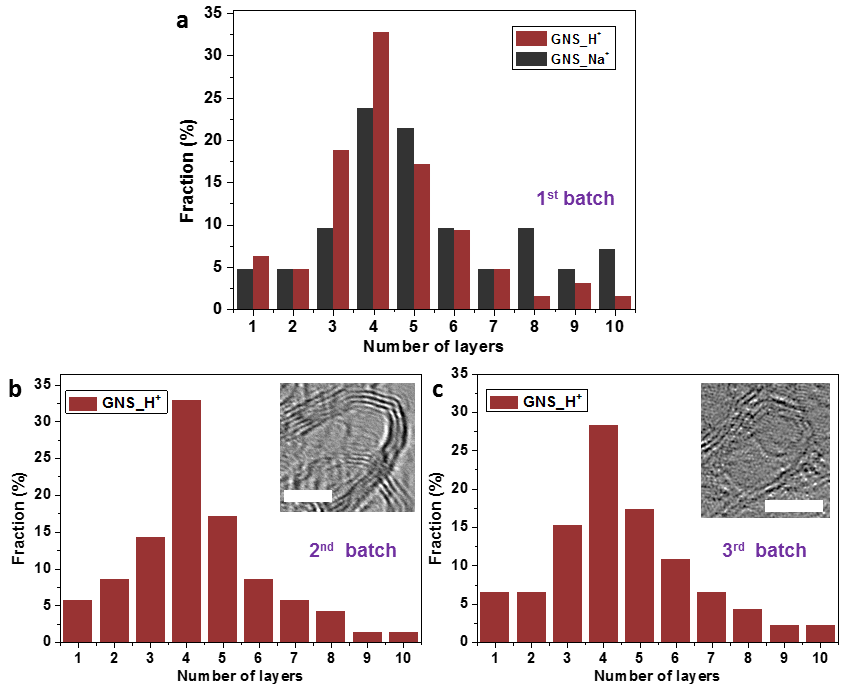
**

**Figure S1** (a) the thickness distribution of more than 30 GNS determined from HRTEM of GNS_Na^+^ and GNS_H^+^(the 1^st^ batch); (b) and (c) are GNS_H^+^ produced from the 2^nd^ and the 3^rd^ batch of the cathodic plasma exfoliation, respectively (scale bar: 4nm).





**Figure S2** (a) XPS survey spectra of GNS_Na^+^ and GNS_H^+^; (b) XRD diffraction patterns of graphite, GNS_Na^+^ and GNS_H^+^; inset: corresponding expanded region of the plots.

**Table S1** Synthesis methods, times, and temperatures; specific surface areas; and electrochemical performances, in NaCl solution at various scan rates, of graphene materials reported in the literature. *rt: Room temperature

| Electrode material | Method | Process time /temperature | Specific surface area (m^2^ g^–1^) | Scan rate (mV s^–1^) | Specific capacitance (F g^–1^) | Ref. |
| --- | --- | --- | --- | --- | --- | --- |
| 3D graphene  sponge | Freeze drying+annealing | 123 h/–53 and 800 °C | 356 | 5 | 205.2 | [1] |
| 3D graphene | Sacrificed template | 42 h/650 °C | 824 | 1 | 151.7 | [2] |
| Sponge-templated  graphene | Sponge-template | 2 h/900 °C | 305 | 10 | 57 | [3] |
| 3D macroporous graphene | Sacrificed template | 6 h/900 °C | 339 | 5 | 58.4 | [4] |
| 3D porous graphene | KOH-activation | 49 h/800 °C | 3513 | 2 | 20.1 | [5] |
| GNS_H^+^ | Cathodic plasma | <1 h/rt* | 464 | 5 | 67.1 | This work |
| GNS_Na^+^ |  |  | 72 | 5 | 19.9 |  |





**Figure S3** (a-c) Cyclic voltammograms and (d-f) galvanostatic charge/discharge curves of (a, d) graphite, (b, e) GNS_Na^+^ and (c, f) GNS_H^+^, recorded at various scan rates.


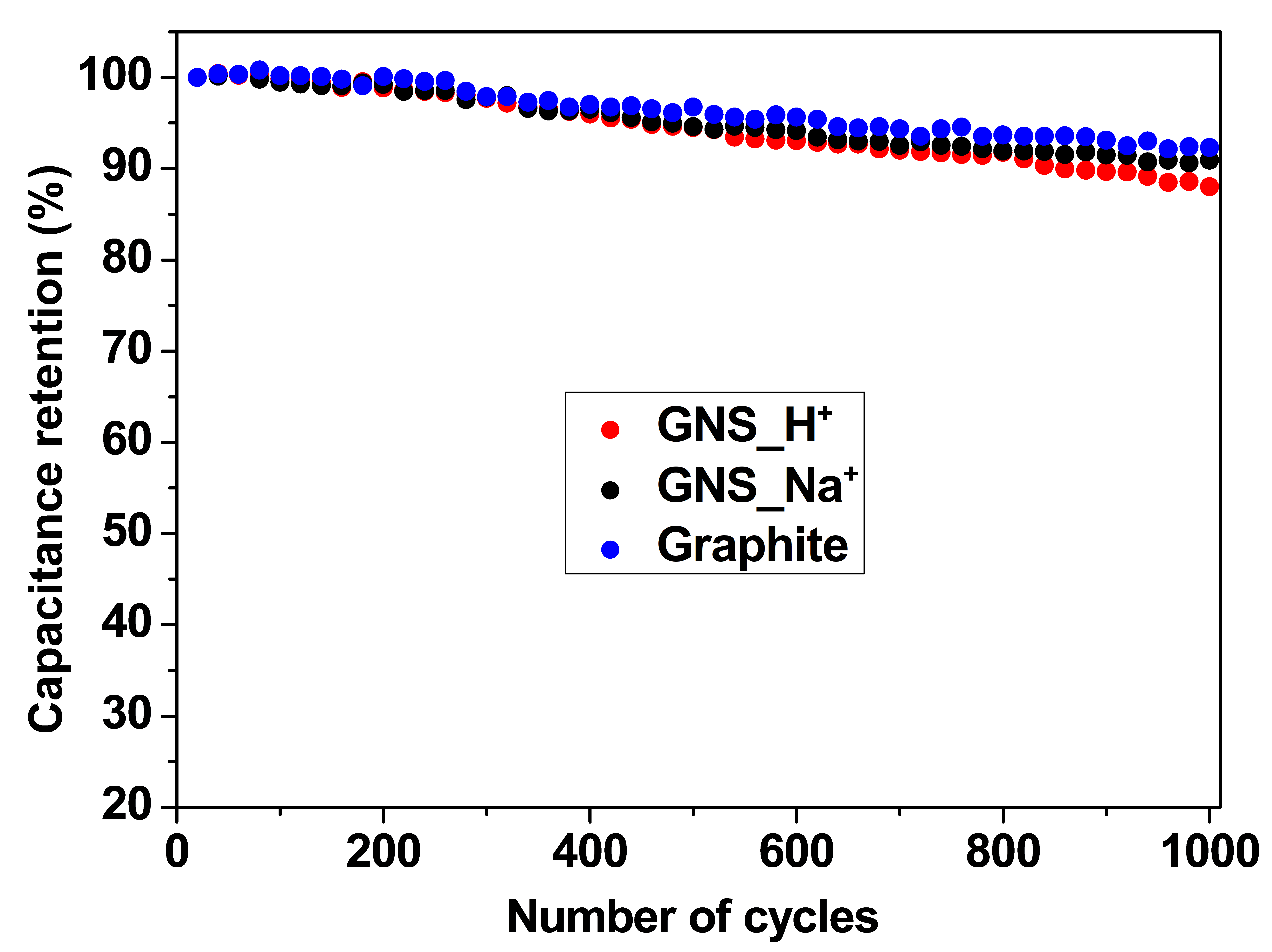


**Figure S4** Cycling tests of graphite, GNS_Na^+^ and GNS_H^+^ over 1000 cycles at a scan rate of 100 mV s^–1^.

**References**

1. Xu X, Pan L, Liu Y, et al (2015) Facile synthesis of novel graphene sponge for high performance capacitive deionization. Sci Rep 5:8458. https://doi.org/10.1038/srep08458

2. Wang H, Yan T, Liu P, et al (2016) In situ creating interconnected pores across 3D graphene architectures and their application as high performance electrodes for flow-through deionization capacitors. J Mater Chem A 4:4908–4919. https://doi.org/10.1039/C5TA10703B

3. Yang Z-Y, Jin L-J, Lu G-Q, et al (2014) Sponge-Templated Preparation of High Surface Area Graphene with Ultrahigh Capacitive Deionization Performance. Adv Funct Mater 24:3917–3925. https://doi.org/10.1002/adfm.201304091

4. Liu X, Wu Y, Yang Z, et al (2015) Nitrogen-doped 3D macroporous graphene frameworks as anode for high performance lithium-ion batteries. J Power Sources 293:799–805. https://doi.org/10.1016/J.JPOWSOUR.2015.05.074

5. Li Z, Song B, Wu Z, et al (2015) 3D porous graphene with ultrahigh surface area for microscale capacitive deionization. Nano Energy 11:711–718. https://doi.org/10.1016/J.NANOEN.2014.11.018
